# Implementing the QUALI-DEC project in Argentina, Burkina Faso, Thailand and Viet Nam: a process delineation and theory-driven process evaluation protocol

**DOI:** 10.1080/16549716.2023.2290636

**Published:** 2023-12-22

**Authors:** Amanda Cleeve, Kristi Sidney Annerstedt, Ana Pilar Betrán, Helle Mölsted Alvesson, Charles Kaboré Wendyam, Guillermo Carroli, Pisake Lumbiganon, Mac Quoc Nhu Hung, Karen Zamboni, Newton Opiyo, Meghan A. Bohren, Soha El Halabi, Celina Gialdini, Mercedes Vila Ortiz, Ramón Escuriet, Michael Robson, Alexandre Dumont, Claudia Hanson

**Affiliations:** a Department of Women’s and Children’s Health, Karolinska Institutet, and Karolinska University Healthcare facility, Stockholm, Sweden; b Department of Global Public Health, Karolinska Institutet, Stockholm, Sweden; c UNDP/UNFPA/UNICEF/World Bank Special Program of Research, Development and Research Training in Human Reproduction (HRP), Department of Sexual and Reproductive Health and Research, World Health Organization, Geneva, Switzerland; d Institut de Recherche en Sciences de la Santé, Ouagadougou, Burkina Faso; e Centro Rosarino de Estudios Perinatales, Rosario, Argentina; f Department of Obstetrics and Gynaecology, Faculty of Medicine, Khon Kaen University, Khon Kaen, Thailand; g Pham Ngoc Thach University, Ho Chi Minh City, Vietnam; h The Global Fund, Geneva, Switzerland; i Gender and Women’s Health Unit, Centre for Health Equity, School of Population and Global Health, University of Melbourne, Melbourne, Australia; j Faculty of Health Sciences, Fundacio Blanquerna, Barcelona, Spain; k Department of Health, Government of Catalonia, Spain; l The National Maternity Hospital and University College Dublin, National University of Ireland, Dublin, Ireland; m Université Paris Cité, Research Institute for Sustainable Development (IRD), Inserm, Paris, France; n Department of Disease Control, London School of Hygiene and Tropical Medicine, London, UK

**Keywords:** Non-clinical interventions, informed decision-making, evidence-based practices, QUALI-DEC, multifaceted intervention, implementation science, theory of change, maternal health

## Abstract

The project ‘Quality Decision-making by women and providers’ (QUALI-DEC) combines four non-clinical interventions to promote informed decision-making surrounding mode of birth, improve women’s birth experiences, and reduce caesarean sections among low-risk women. QUALI-DEC is currently being implemented in 32 healthcare facilities across Argentina, Burkina Faso, Thailand, and Viet Nam. In this paper, we detail implementation processes and the planned process evaluation, which aims to assess how and for whom QUALI-DEC worked, the mechanisms of change and their interactions with context and setting; adaptations to intervention and implementation strategies, feasibility of scaling-up, and cost-effectiveness of the intervention. We developed a project theory of change illustrating how QUALI-DEC might lead to impact. The theory of change, together with on the ground observations of implementation processes, guided the process evaluation strategy including what research questions and perspectives to prioritise. Main data sources will include: 1) regular monitoring visits in healthcare facilities, 2) quantitative process and output indicators, 3) a before and after cross-sectional survey among post-partum women, 4) qualitative interviews with all opinion leaders, and 5) qualitative interviews with postpartum women and health workers in two healthcare facilities per country, as part of a case study approach. We foresee that the QUALI-DEC process evaluation will generate valuable information that will improve interpretation of the effectiveness evaluation. At the policy level, we anticipate that important lessons and methodological insights will be drawn, with application to other settings and stakeholders looking to implement complex interventions aiming to improve maternal and newborn health and wellbeing.

**Trial registration**: ISRCTN67214403.

## Introduction

Rates of caesarean section have increased steadily worldwide over the last three decades; currently 21% of all births are through CS, with the highest rates found in Latin America and the Caribbean (42.8%) and the lowest in sub-Saharan Africa (5%) [1]. CS can be lifesaving, however, ‘at a population level, CS rates higher than 10% are not associated with reductions in maternal and newborn mortality rates’ [2]. Global CS trends suggest substantial inequalities both within and between countries and geographical regions of the world, in which overuse and unmet needs coexist [3]. The unmet need of CS is especially common in low resource settings and among the poor or socially marginalised, which perpetuates and fuels disparities [1]. Inappropriate use of CS, be it overuse or underuse, bears negative health consequences for women and their babies and may divert scarce resources to those who need them the least [4]. Hence, there is an urgent need to ensure equitable access to CS and call for action for the appropriate use of CS.

Given the increasing role of non-clinical factors in the global rise of CS, non-clinical interventions have been recognized as a promising avenue to optimize CS rates and to promote respectful good-quality care for women [5]. We adapted and implemented a multifaceted intervention, based on the World Health Organization (WHO) guideline recommendations [6,7] aiming to optimize the use of CS, promote informed decision-making about mode of birth, and create an enabling and supportive environment for women giving birth. The intervention includes four evidenced-based non-clinical interventions, which are implemented simultaneously and with a view to work synergistically; 1) opinion leader, 2) audit and feedback for CS, 3) labour companionship, and a 4) decision analysis tool (DAT) [8]. In 2020, the ‘Quality Decision-making by women and providers’ (QUALI-DEC) project was initiated in 32 healthcare facilities across Argentina, Burkina Faso, Thailand, and Viet Nam to implement the above four non-clinical interventions. Given the difficulties in understanding what works and why for complex interventions such as QUALI-DEC, we aim to assess how and why QUALI-DEC brought about change using a theory-driven process evaluation to complement the effectiveness evaluation. The evaluation strategy relating to the effectiveness of the QUALI-DEC intervention was outlined in Dumont et al. [9], which also provided an overview of the key functions and methodology of the process evaluation. In this paper, we describe the implementation process and detail in depth, how we plan to conduct the QUALI-DEC process evaluation, which aims to assess:

1. how and why implementation strategies were contextually adapted prior to and during implementation;
2. if, how and for whom did QUALI-DEC work, and why;
3. the mechanisms of change, as well as their interactions with context and setting;
4. how the intervention with its four components can be optimised and the feasibility of scaling it up; and
5. the cost-effectiveness of the intervention

## Methods/design

### Study design

We will use a mixed-methods approach, guided by the Medical Research Council (MRC) updated framework for evaluating complex interventions [10]. The QUALI-DEC process evaluation is nested within a pragmatic hybrid effectiveness-implementation type III trial, which includes an interrupted time-series-analysis (primary outcome) and a before and after cross sectional study (secondary outcomes) [9]. Below we elaborate on 1) the study settings, 1) what is currently being implemented and how, 3) the theory of change (ToC), 4) indicators to measure level of implementation, and 4) how we plan to conduct the process evaluation. Further, we delineate how the six core elements of context, theory, stakeholder engagement, identification of key uncertainties, ongoing intervention refinement, and economic considerations [10], were considered in the planning of the process evaluation. As such, this paper should be viewed as in-depth elaboration the QUALI-DEC study protocol [8], in which we go from generics to specifics, honing in on the process evaluation. We report the protocol according to The Standards for Reporting implementation Studies (STaRI) checklist [11].

### Study settings and sites

All countries include teaching healthcare facilities and a mix of healthcare facilities at tertiary and secondary levels of care. Two out of the 32 healthcare facilities are private, however, 12 of the included public healthcare facilities have private wards. Data collected during 2020, as part of the formative research phase (see below), showed how caseloads, CS rates and infrastructure differs between study countries and healthcare facilities (Table 1). Details on key health indicators by country are presented elsewhere [9].

**Table 1.** Caseload and infrastructure per country; 8 healthcare facilities per country, between January to December 2020.

Item	Argentina	Burkina Faso	Viet Nam	Thailand
Total births (any mode of birth)	12 214	31 144	85 129	32 363
CS; n(%)	4 430 (36; range 30–42%)	9 765 (31; range 15–48%)	45 234 (53; range 24–82%)	14 858 (46; range 34–56%)
Beds for active labour; n (mean, SD)	23 (3; 1)	38 (5; 2)	88 (11; 10)	51 (6; 6)
Facilities for labour and birth	Shared* (*n* = 6)	Shared (*n* = 8)	Shared (*n* = 6)	Shared (*n* = 7)
	Private (*n* = 2)		Both shared and private (*n* = 2)	Private (*n* = 1)
Cardiotocography	Available in all healthcare facilities, 2–9 per healthcare facility	Available in 3 healthcare facilities, 1 per healthcare facility	Available in all healthcare facilities, 4–51 per healthcare facility	Available in all healthcare facilities, 9–20 per healthcare facility
Medical records in healthcare facilities	Electronic and paper (*n* = 8)	Paper (*n* = 8)	Electronic (*n* = 5); Paper (*n* = 2), both electronic and paper (*n* = 1)	Electronic (*n* = 3); paper (*n* = 5)

*Shared during labour but private rooms available when the woman gives birth.

### QUALI-DEC intervention

This multifaceted intervention comprises four non-clinical interventions: 1) opinion leaders to implement clinical algorithms and guidelines and improve adherence to best clinical practices; 2) CS audits and feedback using the Robson classification [12]; 3) a decision-analysis-tool for women to enable informed and shared decision-making for mode of birth; and 4) labour companionship to support women during labour and birth. The selection of the four interventions were guided by results from previous randomised controlled trials with moderate- to high-certainty evidence supporting their effectiveness [7,9,13].

### Implementation strategy

#### Formative research and preparation phase

In line with the updated MRC framework [10] the QUALI-DEC intervention is underpinned by the understanding of *context* as multidimensional and a core element in all phases of the project. A formative research phase preceding implementation was conducted between November 2019 and May 2020, focusing on understanding our study contexts [14–17], including health system readiness, regulatory environment, clinical practice, and socio-cultural values. Implementing partners in participating countries, and supporting partners not directly involved in implementation, conducted research including 1) a document review where the teams in Argentina, Burkina Faso, Viet Nam and Thailand collected information on health system structures such as legal aspect and financing, 2) a readiness assessment of all included healthcare facilities to understand workload, staffing, space, equipment, existing clinical practice guidelines and supplies, and 3) qualitative in-depth interviews with women, their potential companions, healthcare providers and administrators. In Argentina, the formative research phase built on work conducted prior to the initiation of QUALI-DEC [18,19]. Project timelines per country are presented in supplementary Table S1.

We analysed contextual factors in the four countries to assess the societal and institutional factors contributing to inappropriate use of CS in included study healthcare facilities and to identify main barriers of and facilitators to the implementation of the four intervention components. We also used this formative phase to collect baseline data on clinical practice and maternal and neonatal outcomes and CS rates in each of the participating healthcare facilities. CS rates were collected monthly for at least a year prior to implementation, starting in January 2020. We used a cross sectional survey among a representative sample of postpartum women (postpartum survey I) combined with information from the medical records to collect baseline data. The same survey will be used to collect endline data (postpartum survey II) at the end of the implementation phase.

Prior to the implementation phase, each country started a process of identifying, selecting, and adapting clinical algorithms relating to management of labour and decision-making for CS, with the aim to standardising care practices. These algorithms are used to guide the audit and feedback component of the intervention. Country Principal Investigators (PIs) and opinion leaders were free to select or design clinical algorithms most relevant for their context. However, they were encouraged to reach consensus and use the same algorithm within each country. The WHO guidelines on intrapartum care [20], were one of the sources available to inform and adapt clinical algorithms. The process of identification, adaptation, and adoption of clinical algorithms was iterative. Focused on the country level, it involved discussions between PIs, OLs and healthcare providers within maternity teams in each healthcare facility directly involved in implementation, as well as supporting partners (indirectly involved in implementation). In addition, policies on labour companionship and the DAT were adapted to each study setting during this time period.

The identification of implementation strategies was informed by the findings of the formative research and guided by the COM-B model of behaviour change [21]. This process helped implementers ensure that some key barriers to implementation identified during the formative research phase were addressed. A flexible approach was taken in terms of contextual adaptations of the implementation of the four components, both prior to and during implementation, led by country PIs and opinion leaders. So, while *what* is implemented remains the same across all study settings, *how* the intervention components are implemented and how *much* (i.e. the dose), may vary across and within countries responding to contextual factors and adding another layer of complexity. Key implementation activities applicable to all countries and participating healthcare facilities are presented in supplementary Table S2.

#### Implementation phase

We defined the start of implementation as the completion of a 5-day stakeholder training, which aimed to introduce and practice intervention tools and techniques and to increase the confidence and competence of opinion leaders in: conducting caesarean audit; using and interpreting the Robson Ten Group Classification System (RTGCS) [12] and clinical algorithms; applying the DAT; and implementing labour companionship. Country PIs were involved in the development and adaptation of training materials used during the stakeholder training to ensure they were contextually appropriate to meet local needs. also involved healthcare providers within the maternity teams, tasked with supporting the opinion leaders, collecting monthly CS rates, assuring data quality, and applying the RTGCS. Due to the COVID-19 pandemic, the stakeholder training and start of implementation differed between countries (supplementary Table S1). The intervention is implementation over two years and the opinion leaders and their teams of healthcare providers, receive financial incentives to conduct regular audits during this period and implement the intervention.

#### Interventions

##### Opinion leaders and audit and feedback

Opinion leaders are clinicians or hospital administrators with a reputable influence in their workplaces and good communication skills. Following the formative research phase, country PIs identified 1–2 opinion leaders at each participating healthcare facility. In addition, some countries have appointed a ‘coordinator’ role who function as a mentor to the opinion leaders. In all participating healthcare facilities, opinion leaders were tasked with supporting and facilitating the implementation of the three intervention components (Audit and feedback, Companionship, and the DAT), including the clinical algorithms, hence, they are at the centre of this intervention, driving implementation.

Following the stakeholder training, opinion leaders in each healthcare facility established a local QUALI-DEC committee overseeing the implementation and created an audit committee for CS in charge of the audits and feedback sessions. Audit committees are multidisciplinary with representatives of the different professional cadres that are present within the maternity teams as well as healthcare facility administration. The QUALI-DEC protocol specifies that the audit committees should conduct monthly audits of all or a randomised number of CS in lower-risk women (groups 1–4 in RTGCS) and feedback the results and recommendations to the maternity teams through meetings and by displaying the results on dashboards. To enable CS audits, monthly data on CS rates are collected in all participating healthcare facilities and organised according to the RTGCS.

Opinion leaders are free to support implementation as needed in their context. One way employed by opinion leaders is to provide on-site training opportunities on how to apply clinical algorithms, how to use and interpret the RTGCS, and the management of vaginal labour. Opinion leaders assess the needs of their colleagues and organise training sessions and workshops to meet these needs. This means that the type of training, amount and timing of training provided to healthcare providers may vary between participating healthcare facilities and study settings.

##### Decision analysis tool and labour companionship

Opinion leaders also facilitate the implementation of the DAT [8] in antenatal care within the healthcare facilities and, where feasible, within connected primary antenatal care facilities. The paper-based DAT booklet or its electronic version (mobile application for smartphones and Android) is introduced in the third trimester of pregnancy to facilitate the discussion about mode of birth. However, some participating healthcare facilities introduce the DAT earlier due to feasibility in the specific clinical setting. When the DAT is introduced, the pregnant woman is also informed about the option of having a labour companion. Concurrently, the opinion leaders support the implementation of labour companionship within the healthcare facilities, for instance by addressing logistical issues or raising awareness about its benefits, and by strengthen skills in the management of vaginal birth among maternity teams. The role of the opinion leader in terms of supporting and facilitating labour companionship differs depending on 1) how much experience already existed within the healthcare facility’s pre-implementation, and 2) the structural and logistical solutions required to integrate this intervention into routine practice.

##### Communication

Each country has developed their own information, education, and communication (IEC) strategies for internal communication within healthcare facilities and for external communication with professional organisations, policymakers, parliamentarians, and communities-at-large. These strategies were locally developed and target different audiences. Internal communications include booklets, posters and QR codes, aiming to raise awareness of the intervention among healthcare providers and pregnant women visiting the healthcare facilities and antenatal clinics. External communications consist of social media campaigns in Argentina, Thailand and Vietnam, and awareness raising within learning institutions.

### Process evaluation

#### Theory of change

**Figure 1.**
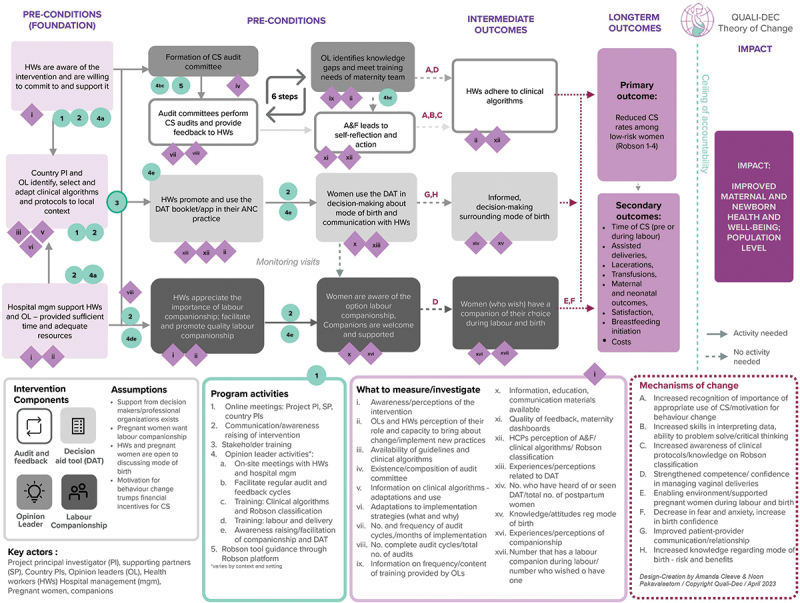
QUALI-DEC Theory of Change.

After the formative research, implementing partners in participating countries, each drafted a country-specific Theory of Change (ToC) [22] which envisaged how the QUALI-DEC intervention might achieve change in their settings. Findings from the formative research phase and the countries’ ToC were utilised on a country level to help adapt the implementation of the four components of the intervention during a stakeholder meeting. The first step in the development of the QUALI-DEC process evaluation was to develop a ToC applicable across all countries. This was done through discussions in the project team and the using country level ToC as a point of departure. The ToC was presented during an in-person meeting in September 2022, whereby it was further refined, guided by De Silva et al. [22] for the integration of theory in the MRC updated framework [10] (Figure 1). We used the ToC to guide the process evaluation including which questions to ask and perspectives to seek. The ToC specifies the intervention pre-conditions [22] i.e. the necessary links in the causal pathway for the project to achieve its impact. We used intervention pre-conditions to identify key uncertainties [10]. Given what is known about the intervention [9] and study settings, key uncertainties represent the ‘unknown’ and the most important aspects to uncover in the project evaluation. They may relate to questions about how the intervention components were implemented or what the hypothesised mechanisms of change were. The identification of key uncertainties was informed by observations by implementing partners directly involved in implementation activities, which included on-the-ground implementation perspectives. Key uncertainties were subsequently used to develop prioritised research questions for the process evaluation. These questions and their corresponding data sources are presented in Table 2, which also shows the linkage between the intervention pre-conditions and key uncertainties – research questions.

**Table 2.** Key research questions for the process evaluation with corresponding pre-conditions and data sources.

Intervention pre-condition [22] as shown in ToC, Figure 1.	Key research questions	Source
**Overall**		
●Country PIs and selected opinion leaders identify, select, and adapt clinical algorithms and protocols to local context	Which adaptations were done to implementation strategies and/or context to implement the complex intervention? When were adaptations done and why?	Formative research, stakeholder engagement reports, monitoring visits, monthly meetings
●Health workers are aware of the intervention and are willing to commit to and support it ●Healthcare facility management supports health workers and opinion leaders; they are provided sufficient and adequate resources	What were the key internal and external factors impacting motivation, morale, capacity for behaviour change among healthcare providers?	Survey evaluating the stakeholder training Interviews with healthcare providers and opinion leaders
N/A	Is QUALI-DEC cost-effective?	Monitoring visits, financial and project reports, the postpartum survey, and interviews with project staff
**Opinion leader**		
●Country PIs and selected opinion leaders identify, select, and adapt clinical algorithms and protocols to local context. ●Formation of audit committee ●OLs identifies knowledge gaps and meet training needs in maternity team.	How was the intervention Opinion leader implemented? What was the quality and reach of the opinion leader intervention?	Process/outcome indicators (table 4) Interviews with opinion leaders
	What were opinion leaders’ perceptions of their own role and capacity to bring about change (in relation to the 3 other intervention components)?	
**Audit and feedback**		
●Country PIs and selected opinion leaders identify, select, and adapt clinical algorithms and protocols to local context. ●Audit committees perform CS audits and provide feedback to Health workers. ●Audit and feedback lead to self-reflection and action	How was the intervention Audit and Feedback implemented? What was the quality of the Audit and feedback?	Process/outcome indicators (table 4) Interviews with healthcare providers and opinion leaders
	How did healthcare providers understand and engage with the RTGCS and related clinical algorithms?	
	How were the audit and feedback sessions perceived by opinion leaders and health workers?	
	Did feedback lead to increased adherence to clinical algorithms? Why? Why, not?	
**Labour companionship**		
●Country PIs and selected opinion leaders identify, select, and adapt clinical algorithms and protocols to local context. ●Health workers appreciate the importance of labour companionship; they facilitate and promote quality labour companionship. ●Pregnant women are aware of the option labour companionship, companions are welcome and supported	How was labour companionship implemented and adapted in participating healthcare facilities?	Process/outcome indicators (table 4) Interviews with healthcare providers and postpartum women Postpartum survey II
	What was the quality and reach of the labour companionship intervention?	
	How was companionship perceived and experienced by women, companions, health workers and healthcare facility management?	
**Decision analysis tool**		
●Country PIs and selected opinion leaders identify, select, and adapt clinical algorithms and protocols to local context	How/where was the DAT implemented in participating healthcare facilities and in primary healthcare? What was the reach of the DAT?	Process/outcome indicators (table 4) Postpartum survey II Interviews with healthcare providers and postpartum women
●Health workers promote and use the DAT booklet/app in their ANC practice, communication material in relevant places. ●Pregnant women use the DAT booklet/app in their CS decision-making and communication with HCP	How did health workers and women engage with and perceive the use of the DAT including its impact on the patient provider-relationship and decision-making?	

The QUALI-DEC project ToC illustrates the logical causal pathway for the intervention. Each precondition in the causal pathway is essential for the long-term outcome and impact to be achieved and is linked to specific project activities. In turn, the project activities illustrate *how* we will reach our outcomes. Each outcome is linked to an item representing *what we will measure and document* to know whether we are making progress towards each outcome. Intervention assumptions reflect external conditions that are beyond the control of the project that must exist for our outcomes to be achieved [22]. Although assumptions are listed as applying to the project as a whole, we presume that there are contextual differences both between countries and within countries, which make some assumptions more and sometimes less relevant. We expect maternal and newborn health and well-being to improve as adherence to clinical algorithms increase and rates of CS subsequently decrease in low-risk women (RTGCS 1–4), vaginal births increase, and satisfaction with labour care increase. Further, we foresee that the project’s long-term outcome –, appropriate use of CS – measured through the primary and secondary outcomes, will lead to our impact.

The intervention components are anticipated to produce change synchronically and synergistically. The synergistic nature of the intervention is illustrated in the ToC; it means that the way in which intervention components relate and interact will amplify the effect of the individual intervention components. *Opinion leaders* have central functioning and are expected to contribute to our outcomes directly and indirectly, directly through the training and support of healthcare providers, and indirectly through awareness raising and facilitation of other intervention components, including the formation of the Audit committee. Opinion leaders are expected to facilitate the implementation of all intervention components, however, the specific actions and facilitation activities required are contextual. This means that the size of the effect of opinion leaders and their interaction with other components, may vary by context and setting.

*Audits and feedback* are anticipated to lead to behaviour change by encouraging reflection and analysis of own and fellow healthcare providers’ practices. We anticipate that the effect of audits and feedback will be threefold; greater awareness about the importance of appropriate use of CS, increased ability to interpret clinical data, and increased adherence to guidelines into practice. Furthermore, as adherence to clinical algorithms increases, we expect that feedback will lead to positive reinforcement of this behaviour.

*Labour companionship* is expected to lead to fewer CS conducted after labour has begun, increased rates of vaginal births. In turn, we anticipate that satisfaction with labour care will increase as well as the chances of a subsequent pregnancy ending in a vaginal birth. This component will be strengthened through opinion leaders and the DAT, which raises awareness about labour companionship.

The *DAT* is expected to produce change by increasing knowledge surrounding childbirth among pregnant women; improving the patient-provider relationship; and by promoting a respectful and constructive dialogue. This will enable women to make informed decisions about mode of birth.

IEC strategies are hypothesised to influence awareness of the DAT and labour companionship among pregnant women and subsequently increase uptake of these intervention components. As part of the evaluation process, we aim to document the specifics of the local IEC strategies but will not attempt to evaluate their impacts.

#### Process evaluation data sources

We will use multiple data sources in our process evaluation, measured and collected at different time points, to understand what worked, for whom it worked and why. Main data sources are 1) quarterly monitoring visits, 2) evaluation of the stakeholder training, 3) the before and after cross sectional survey among post-partum women, and 4) qualitative interviews with opinion leaders, healthcare providers, and postpartum women.

##### Monitoring visits

Regular monitoring visits are conducted during the implementation phase in each of the participating healthcare facilities using a standardised checklist. The purpose of these visits is to 1) ensure that standard operating procedures are being followed; 2) capture adaptations to implementations strategies; 3) identify and document implementation bottlenecks; 4) document and report on study progress; and 5) capture cost-related information. Data from the monitoring visits will be used to create implementation indicators (see below).

##### Evaluation of the stakeholder training

We will use the results from an online survey to evaluate the 5-day stakeholder training. A survey was sent out to all who participated in training,1–2 weeks prior to the training, immediately after each day of the training, and one week following completion of the training. These surveys sought to understand the impact of training on participants expectations, appreciation of the intervention and its different components, perceptions on barriers and facilitators to implementation, and satisfaction with the different sessions in the stakeholder training, including met needs. Results from the online survey will be a valuable source as the 5-day stakeholder training lays the foundation for implementation.

##### Postpartum survey

To evaluate QUALI-DEC we will use information from two cross-sectional postpartum surveys; one was conducted before implementation start and one will be conducted towards the end of the intervention. The postpartum survey comprises face-to-face interviews with a representative sample of postpartum women who gave birth in participating healthcare facilities during a specific time period. The survey data is linked to information obtained from each woman medical record. The survey provides information on birthing women’s background characteristics; knowledge about and preferences for mode of birth both before and after birth; maternal and neonatal health outcomes; birth experience and satisfaction; and experiences with labour companionship and the DAT. In addition, the survey captures outcomes related to gender and social equity, wealth index and out-of-pocket expenditures. The second postpartum survey will be conducted towards the end of the implementation period, defined as 24 months after the 5-day training (see supplementary Table S1). Information from the postpartum survey will be used to develop implementation indicators (see below).

##### Process and output indicators

Data collected through monitoring visits and the postpartum survey II will be used to gather data relating to our pre-defined process and output indicators capturing information on process evaluation domains, shown in supplementary Table S3. Based on information collected during monitoring visit and consultations with implementing partners, these indicators will be used to establish an implementation score that allows for comparisons across included healthcare facilities and countries. We will score separately each intervention component and each healthcare facility, with a higher composite overall score representing a higher level of implementation. Healthcare facilities will be categorised as having a low, moderate, or high level of implementation. Two people will independently score each monitoring visit and any conflicts will be arbitrated by the country PI to ensure the quality of the scoring process.

##### Interviews with opinion leaders

Individual in-depth interviews (IDIs) will be held with opinion leaders in all participating healthcare facilities towards the end of implementation. These interviews will explore experiences of implementation of the four components and further into depth about the role of the opinion leader and his/her perceptions about this role. Interviews with opinion leaders will also be part of case studies described below.

##### In-depth case studies

We plan to conduct in-depth case studies [23] in two participating healthcare facilities per country towards the end of implementation (Supplementary Table S1). We aim to select better performing healthcare facilities, in terms of how much they have implemented, as we assume that these will be able to provide a richer description of their implementation experiences. To select two better performing healthcare facilities, we will use the indicators generated from the monitoring visit checklist (Supplementary Table S3). We plan to select one healthcare facility with a high score and one with a medium composite score. If in one country, there are no healthcare facilities with both high and medium composite scores, we will aim to select two healthcare facilities where the composite scores differ enough for us to gather information on implementation experiences that detail both implementation bottlenecks and success stories. The scoring will be done after a minimum of three monitoring visits have been conducted and will be discussed with country PIs who will have the final decision in which healthcare facilities are selected.

Our case studies methodology will include: 1) process and output indicators (supplementary Table S3), 2) experiences of implementation among healthcare providers using IDIs, 3) interviews with opinion leaders (described above), and 4) experiences of labour companionship and the DAT among postpartum women using the postpartum survey II (described above) and IDIs. The IDIs with postpartum women will be a sub-sample of respondents in the postpartum survey in the two selected case-study healthcare facilities. Purposive sampling will be conducted to include women with experiences of the DAT and labour companionship for women with both vaginal and caesarean birth.

##### Additional sources

Additional sources of information valuable to the process evaluation include meeting minutes and recordings from monthly project meetings, as well as discussions during the project kick-off meeting in 2022.

#### Adaptations and scalability assessment

Adaptations of the intervention are captured at different time-points of implementation using various data sources including the monitoring visits (Supplementary Table S3). We categorised adaptations into (i) *early adaptations*, those occurring before the start of implementation; and (ii) *late adaptations*, those occurring during implementation in response to bottle-necks and challenges identified or emerging during implementation. Scalability will be assessed at two timepoints, pre-implementation and at the end of implementation, guided by a scalability assessment tool [24]. The tool was developed through a review and synthesis of scale-up framework’s pre-implementation. Information gained from interviews with opinion leaders and healthcare providers, and a synthesis of early and late adaptations, will be used to provide evidence on the feasibility of scaling up QUALI-DEC, with emphasis on which intervention components to scale up and how.

#### Cost evaluation

An extended cost-effectiveness analysis (ECEA) will be used to evaluate the cost-effectiveness of the QUALI-DEC intervention [25]. Data sources to calculate implementation costs will be collected from the monitoring visits, financial and project reports, the post-partum survey I and II and interviews with the project staff. The denominator (health effects i.e. targeted reduction in CS among low-risk women), women’s out-of-pocket costs averted, and financial risk protection will be collected from the before and after post-partum survey.

##### Sample size

We plan to include a total of 940 participants per country in the before and after cross sectional survey among postpartum women, for which the sample size calculations are published elsewhere [9]. The postpartum survey will be carried out in all 32 healthcare facilities. Similarly, we will interview 1 to 2 opinion leaders per study healthcare facility for a total sample of 8 to 16 IDIs per country to obtain the widest possible perspective from all 32 healthcare facilities.

The online survey used to evaluate the 5-day stakeholder training was sent out 3 times to all who took part of the training in each country. The sample size differs between countries, day of the training and training session – for each session in the training there were between 15 to 30 participants.

For IDIs with postpartum women, carried out as part of the case studies, maximum variation sampling will be used to achieve a stratified sample without random selection. This method uses pre-specified parameters to stratify the sample and encourages recruitment and sampling based on diversity [26]. We will recruit 10–12 women per hospital to participate in IDIs, aiming for diversity in terms of experience with the DAT and labour companionship, among women giving birth vaginally and through CS. Further, we will conduct 10–12 IDIs with health workers in each of the two healthcare facilities selected for the case study, with a total sample of 20–24 IDIs per country. Participants will be selected with the aim to include different professional cadres with varying duration of clinical experience, that have been actively involved with the implementation of all or some intervention components.

##### Analysis

Quantitative indicators will be analysed and presented descriptively comparing overall scores across healthcare facilities per individual intervention component in each country and for all intervention components per country. Their interpretation will take into consideration data collected through postpartum survey I and II, findings from the pre-implementation readiness assessments and information gained through qualitative interviews conducted as part of this process evaluation. Qualitative interviews will be analysed using the Framework Method [27]. Analysis of data generated from the postpartum surveys is presented elsewhere [9].

We will use descriptive statistics to analyse the online surveys for the evaluation of the stakeholder training, comparing means with t-test and ANOVA, and proportions using chi-square. With regards to costs, distributional consequences and where appropriate given the context, financial risk protection will be estimated. The impact of QUALI-DEC will be estimated in three domains across wealth index: (i) health gains (e.g. reduced CS rates in RTGCS 1–4), (ii) women’s out-of-pocket (OOP) expenditures averted by reducing specified CS, and (iii) total net cost of the intervention. An incremental cost-effectiveness ratio will be presented to estimate the cost per reduction in targeted CS.

## Discussion

To optimise CS rates and improve equity in access to CS, WHO has emphasised a need more robust evidence on multifaceted non-clinical interventions [7]. In QUALI-DEC, we simultaneously apply four different evidence-based non-clinical interventions targeting women and healthcare providers with implications for health systems, which we believe will have a synergistic effect and ultimately improve maternal and neonatal outcomes [9]. The QUALI-DEC process evaluation detailed in this protocol will provide valuable insights, including how these non-clinical interventions produced change, when and where.

Research has contributed to a better understanding of the myriad of factors contributing to overuse of CS, including women’s preferences for CS and the influence of socio-cultural context on these preferences [28–30]. The situations and circumstances in which women make choices regarding mode of birth, and on what grounds, has been highlighted as key questions that warrant further attention [8,31,32]. The QUALI-DEC intervention combines interventions that directly aim to lower CS rates among low-risk women, and interventions that strive to empower pregnant women and improve childbirth experiences by promoting informed decision-making about mode of birth and creating enabling environments allowing for labour companionship. By increasing adherence to evidence-based clinical algorithms, the intervention also aims for equity in access to CS, addressing both overuse of, and unmet need for, CS. Our process evaluation will provide a better grasp on whether these aims were achieved and, if so, for whom and under what circumstances. In addition, it may lead to improved understanding how certain push and pull factors for CS influence implementation processes and outcomes, such as sociocultural context, women’s preferences, health system structures including financial incentives, and power dynamics between pregnant women and healthcare providers.

Complex interventions such as QUALI-DEC, conducted in diverse study settings, require flexibility in how interventions and implementation strategies are tailored to fit study contexts [33]. Further, barriers to change are often multifaceted and require multiple interventions adapted to setting, the target group and specific obstacles to change [34]. In QUALI-DEC, we have chosen a mixed methods approach to evaluate QUALI-DEC, including case studies [33]. Case studies as a methodology has been criticised for lacking rigour and its findings to have limited generalisability. One point of critique raised is that the selection of only ‘typical cases’ or the ‘wrong cases’ may limit theoretical generalisations of a case study [35]. Concurrently, the case study approach can generate a better understanding of causal mechanisms and the contextual conditions that are necessary for implementation, rendering it especially suitable for evaluations of complex interventions [36]. For the purposes of the QUALI-DEC process evaluation, the inherent methodological flexibility offers an opportunity to explore implementation experiences in-depth, triangulating research methodologies and perspectives. Case studies will be conducted in healthcare facilities that have been able to implement QUALI-DEC intervention components to some extent, and not where implementation bottlenecks have impeded most or all implementation efforts (for reasons provided above). Nevertheless, by interviewing opinion leaders from all participating healthcare facilities, regardless of implementation level, we hope to capture perspectives from healthcare facilities with no or lower levels of implementation. This will enable us to elucidate contrasting voices while generating an in-depth understanding of implementation experiences in selected healthcare facilities, complemented by our effectiveness- and cost-evaluation.

This process evaluation is strengthened by our theory-driven approach. The value of integrating theory in the development and evaluation of complex interventions has gained increasing attention [37] and been described as ‘vital when building an evidence base that informs policy and practice’ by the MRC [33]. Further, integrating theory in design, adaptation, and evaluation, is important to help clarify the relationships between context, mechanisms of change and outcomes and thereby help design more tailored interventions [38]. In QUALI-DEC we have integrated theory from the development stage to evaluation. By making explicit the theoretical assumptions, hypothesised causal pathways and mechanisms of change we hope to facilitate interpretation of the project evaluation and enhance generalisability to other settings [22,37]. In addition, we presume that a theory-driven approach and using a ToC in evaluating QUALI-DEC, is better fit to test synergistic effects of the intervention and to unearth complex or hidden causal pathways [22].

At the policy level, we anticipate that important lessons with application to other similar settings and stakeholders will be drawn from the QUALI-DEC process evaluation, both overall and for specific intervention components. These will be valuable to future implementation endeavours aiming to address over and underuse of CS and improve the quality of labour and childbirth care through non-clinical interventions.

## Supplementary Material

SUPPLEMENTARY TABLES.docx

## Data Availability

The data produced and published during the QUALI-DEC project will be accessible in Zenodo (https://www.zenodo.org/), a general-purpose open access repository developed under the European OpenAIRE program and operated by the European Organization for Nuclear Research (CERN).

## Paper Context

Rates of caesarean sections are steadily increasing worldwide. Many settings are characterised by a complex scenario in which unmet needs and overuse coexist. Our complex intervention combines four non-clinical interventions to optimise caesarean sections rates and to improve maternal and newborn health across 4 countries. Using a mixed methods approach, we plan a theory-driven process evaluation that will generate methodological insights and in-depth understanding of implementation processes and experiences, including information on scalability and cost-effectiveness.
