# Supplementary material for: Implementing the QUALI-DEC project in Argentina, Burkina Faso, Thailand and Viet Nam: a process delineation and theory-driven process evaluation protocol
Source: Glob Health Action. 2023 Dec 22;16(1):2290636. doi: 10.1080/16549716.2023.2290636 (PMC10763892; doi:10.1080/16549716.2023.2290636)
Supplement: SUPPLEMENTARY TABLES.docx [file ZGHA_A_2290636_SM2886.docx]

SUPPLEMENTARY TABLES

**Supplementary table 1.** Implementation timeline per country

| **Year** | Baseline data collection,  postpartum survey I | Pre-implementation adaptations | Stake-holder training* | Implementation: 24 months | Refresher training** | Qualitative data collection for process evaluation | Endline data collection, postpartum survey II |  |
| --- | --- | --- | --- | --- | --- | --- | --- | --- |
| **Argentina** | | | | | | | | |
| 2020 |  |  |  |  |  |  |  | |
| 2021 |  |  |  |  |  |  |  | |
| 2022 |  |  | Q2 | Q3-4 |  |  |  | |
| 2023 |  |  |  |  | Q2 |  |  | |
| 2024 |  |  |  | Q1-2 |  | Q2 | Q2 | |
| **Burkina Faso** | | | | | | | | |
| 2020 |  |  |  |  |  |  |  | |
| 2021 |  |  | Q3 | Q3-4 |  |  |  | |
| 2022 |  |  |  |  | Q3 |  |  | |
| 2023 |  |  |  | Q1-3 |  | Q3 | Q3 | |
| 2024 |  |  |  |  |  |  |  | |
| **Thailand** | | | | | | | | |
| 2020 |  |  |  |  |  |  |  | |
| 2021 |  |  | Q4 |  |  |  |  | |
| 2022 |  |  |  | Q1^a^-4 |  |  |  | |
| 2023 |  |  |  |  | Q1 |  |  | |
| 2024 |  |  |  | Q1 |  | Q1 | Q1 | |
| **Viet Nam** | | | | | | | | |
| 2020 |  |  |  |  |  |  |  | |
| 2021 |  |  |  |  |  |  |  | |
| 2022 |  |  | Q2 | Q3-4 |  |  |  | |
| 2023 |  |  |  |  | Q2 |  |  | |
| 2024 |  |  |  | Q2 |  | Q2 | Q2 | |

* Completion marks the start of the implementation phase; ** 12 months from completion of stakeholder training;  ^a^Stakeholder training was done in December 2020 but implementation started in March 2021

**Supplementary table 2**. Key project activities applicable to all participating healthcare facilities and countries

| **Time frame** | **Activities** |
| --- | --- |
| Pre-implementation phase | |
|  | Document review and adaptations of clinical protocols including protocols on CS and related algorithms, labour management and companionship* |
|  | Identification of opinion leaders |
|  | Creation of an e-platform to facilitate feedback of CS rates to healthcare providers |
|  | Contextual adaptations to companionship policies and the DAT |
| Implementation phase | |
|  | Stakeholder training of opinion leaders and healthcare providers involved in data collection on CS |
|  | Formation of the audit committees |
|  | Implementation of clinical protocols including protocols on CS and related algorithms, labour management and companionship |
|  | Monthly data collection of CS rates and classification according to RTGSC |
|  | Regular audit and feedback cycles |
|  | Implementation of the DAT in participating healthcare facilities as well as in antenatal care clinics connected to participating healthcare facilities where feasible |
|  | Implementation of labour companionship |
|  | Quarterly monitoring visits |
|  | Refresher training for opinion leaders and data collectors |
|  | Communication activities to support implementation |

* This work was initiated during the stakeholder training in some countries

**Supplementary table 3.** Indicators measuring level of implementation.

| **No.** | **Indicator** | **Process evaluation**  **dimension** | **Answer options and scoring** | **Source** |
| --- | --- | --- | --- | --- |
|  | **Opinion leader and clinical practice guidelines** | | |  |
| 1 | List of members of the local QUALI-DEC (including audit committee) committee available, including positions / titles | Fidelity and quality | 0 = No  1 = Yes | Monitoring visit checklist |
| 2 | Conduct of local QUALI-DEC Committee meetings, attendance list, reports available, including information on frequency and duration of meetings | Fidelity and quality | 0 = No  1 = Yes |  |
| 3 | Clinical algorithms for CS decision-making available | Fidelity and quality | 0 = No  1 = Yes |  |
|  | Range of scores: 0 to 3; 0 = Low; 1-2 = Medium; 3 = High | | | |
|  | **Audit and feedback** | | |  |
| 4 | Conduct of audit cycles by local audit committee according to adapted local schedule | Fidelity^a^ and dose | 0 = No  1 = Yes | Monitoring visit checklist |
| ~~5~~ | Verification of conduct of six standardized steps of each audit cycle from audit reports | Fidelity and quality | 0 = Not done  1 = Partially done  2 = Fully done |  |
| 6 | Audit recommendations implemented | Quality | 0=Not at all  1=No recommendation  2=To some extent  3=Yes, fully |  |
| 7 | Monthly maternity dashboard (e.g., graphs showing distribution of deliveries across Robson groups) available | Fidelity^a^ and dose | 0 = No  1 = Yes |  |
|  | Range of scores: 0 to 7; 0-3 = Low; 4-5 = Medium; 6-7 = High | | | |
|  | **Decision analysis tool (DAT)** | | |  |
| 8 | DAT booklets available in waiting area, antenatal clinic, doctors, nurses’ stations, or other location | Fidelity^a^ and reach | 0 = No  1 = Yes | Monitoring visit checklist |
| 9 | DAT booklet administered to appropriate participants |  | 0 = No  1 = Yes |  |
|  | Range of scores Monitoring visits: 0-2; 0 = Low; 1= Medium; 2= High | | | |
| 10 | Number of postpartum women who have seen or heard of the DAT booklet or app/total number of postpartum women | Reach | 0=none  1=<25%  2=25-75%  3=>75% | Postpartum survey II |
|  | Range of scores postpartum survey II: 0-3; 0-1= Low; 2=2 Moderate; 3=3 High | | | |
|  | **Labour companionship** | | |  |
| 11 | Guidelines or protocols for labour companionship available | Fidelity and quality | 0 = No  1 = Yes | Monitoring visit checklist |
| 12 | IEC materials available in relevant areas | Reach | 0 = No  1 = Yes |  |
|  | Range of scores monitoring visits: 0 to 2; 0 = Low; 1= Medium; 2= High | | | |
| 13 | Number of PP women who had a companion of choice/number of postpartum women who wished to have a companion | Quality | 0=None  1=<25%  2=25-75%  3=>75% | Postpartum survey |
| 14 | Number of postpartum women who had a companion at any time during labour or birth (vaginal or CS)/number of women who wished to have a companion (vaginal birth or in-labour CS) | Reach and coverage | 0=None  1=<25%  2=25-75%  3=>75% | Postpartum survey II |
|  | Range of scores postpartum survey II: 0-6; 0-2= Low; 3-4= Moderate; 5-6= High | | | |
|  | *Overall scores across intervention components and indicators (per healthcare facility and monitoring visit)*  Range of scores^b^ 0-23  Interpretation: 1-7 = Low; 8-15 = Medium; 16-23 = High | | | |

^a^ Fidelity to adapted protocol; ^b^Three levels of implementation defined proportionately by dividing total scores into tertials.
